# The Association Between Access Block And Ambulance Ramping, And The Impact of COVID‐19: A Retrospective Observational Cohort Study of 25 Queensland Hospitals

**DOI:** 10.5694/mja2.70141

**Published:** 2026-02-10

**Authors:** Hwan‐Jin Yoon, Justin Boyle, Ibrahima Diouf, Emma Bosley, Andrew Staib, Vahid Riahi, Hamed Hassanzadeh, Mahnaz Samadbeik, Clair Sullivan, Sankalp Khanna, James F. Lind

**Affiliations:** ^1^ Australian e‐Health Research Centre, CSIRO Melbourne Victoria Australia; ^2^ Australian e‐Health Research Centre, CSIRO Brisbane Queensland Australia; ^3^ Queensland Ambulance Service Brisbane Queensland Australia; ^4^ Princess Alexandra Hospital Brisbane Queensland Australia; ^5^ Queensland Digital Health Centre University of Queensland Brisbane Queensland Australia; ^6^ Gold Coast University Hospital Gold Coast Queensland Australia

**Keywords:** delivery of healthcare, emergency medicine, emergency services, evidence‐based medicine, health policy, health services research, health systems, hospitals, medical, statistical models

## Abstract

**Objective:**

To explore the characteristics of ambulance ramping and its association with access block before, during and after the first wave of the coronavirus disease 2019 (COVID‐19) pandemic.

**Design:**

Retrospective observational study.

**Setting:**

Exploratory data analysis and statistical modelling covering the ambulance–emergency department (ED) interface of the 25 largest public hospitals in Queensland between 1 January 2018 and 31 December 2022.

**Main Outcome Measures:**

Primary outcome: The association between ramping, assessed as the ambulance performance target patient off‐stretcher time (POST) and access block, and how COVID‐19 affected these time‐sensitive processes. Secondary outcomes: The association between POST and ambulance response time and between ramping and ED length of stay.

**Results:**

A significant decline in POST performance was observed across the study period, with the mean difference between pre– and post–COVID‐19 periods being 13.1 min (95% CI, 12.9–13.3 min) and 8.9 min (95% CI, 8.7–9.1 min) for Priority 1 and Priority 2 responses, respectively. POST compliance within 30 min dropped from 74% (718,912) pre–COVID‐19 to 66% (694,633) during the first wave of COVID‐19 and 57% (309,815) post–COVID‐19, all below the 90% target. The proportion of patients experiencing access block increased from 10% (91,168) to 17% (87,757) over this same time period. Regression analyses revealed a positive relationship between POST and access block, response time and POST, and ramping and ED length of stay. Before COVID‐19, no significant relationship existed between POST and access block for triage category 1 patients, but longer POST was linked to a higher likelihood of access block for categories 2–5. This trend increased across all categories during and post–COVID‐19.

**Conclusion:**

Achieving the POST target of transferring 90% of patients within 30 min is becoming more difficult, with performance declining. The strong association of POST with access block suggests that access block is driving ramping increases. To reduce delays, efforts should focus on improving access to ward beds and managing hospital capacity issues.

## Plain Language Summary

**The Known:**

Delays in offloading ambulance patients into emergency departments (EDs) have increased dramatically in recent years and are frequently politicised.

**The New:**

To our knowledge, this study of the ambulance–ED interface at 25 hospitals is the first to explicitly model the relationship between access block and ambulance performance and reveals a strong association between ramping and access block. Shorter length of stay in the ED is associated with reduced ambulance ramping.

**The Implications:**

Ambulance ramping (offload delays) indicates access block issues within hospital systems, not delays in ED operations. Addressing ramping requires improving ward bed access and overall hospital capacity.

## 1 Introduction

Emergency department (ED) presentations are increasing globally [1, 2, 3, 4, 5] and within Australia [6]. ED crowding and congestion are increasingly common issues facing acute healthcare systems internationally and have been associated with negative patient and staff outcomes [7]. Access block, the inability to move admitted patients from the ED to an inpatient bed, is a primary cause of ED crowding [8, 9] and a problem for the entire hospital. The emergency healthcare system faces the ongoing challenge of delivering timely, safe and cost‐efficient care to a growing number of patients [10].

Ramping, also known as ambulance offload delay or delay in patient off‐stretcher time (POST), refers to an extended time interval between an ambulance arriving at the hospital and the transfer of care to hospital staff. It has emerged as an issue affecting care quality, patient safety and the ability of paramedics to respond to other emergencies in the community [10, 11, 12, 13, 14, 15].

Previous research has showed a relationship between ambulance numbers, ED occupancy and POST compliance [16], but the relationship between POST and access block is less clear. Understanding relationships between ambulance performance and ED flow metrics can clarify flow barriers at the ambulance–ED interface. Although the impact of the coronavirus disease 2019 (COVID‐19) pandemic on the health system has been well studied, its effect on the ambulance–ED interface has been largely ignored. Reports have emerged of a post–COVID‐19 pandemic increase in the number of ambulance arrivals with delayed handover to hospital care [17], but the relationship between ramping and access block has not been explicitly explored. Improved ED performance during the first wave of COVID‐19 was attributed to reduced hospital occupancy and decreased elective surgery [18]. However, all Australian health jurisdictions are currently overwhelmed by unprecedented demand (> 9 million ED presentations in 2023–2024 [19]), high levels of access block and frequently politicised ramping, and it is paramount to investigate this crucial interface to acute care delivery. This study investigates the relationship between ambulance ramping and both access block and ambulance response time across different phases of the COVID‐19 pandemic.

## 2 Methods

### 2.1 Data Sources, Definitions and Their Relationships

A retrospective cohort analysis was performed on all patients presenting by ambulance to Queensland public hospital EDs from 1 January 2018 to 31 December 2022, using data from Queensland Health's Emergency Department Collection and Queensland Ambulance Service (QAS) data. The study used aggregated hourly data. The QAS, the largest ambulance service in Australia, responds to over 1400 Code 1 emergencies daily. QAS uses response time and POST to evaluate its service effectiveness. This study included the 25 largest public hospitals in the state, encompassing a range of EDs from Australia's busiest to various regional facilities.

Three key metrics were analysed:

- Response time—measured from a Triple Zero (000) call being answered to the first ambulance's arrival at the scene for QAS Priority 1–2;
- POST—the time between ambulance arrival at hospital and the patient being transferred off the QAS stretcher into the ED for QAS Priority 1–2; and
- Access block—the number of patients admitted from the ED who were delayed from leaving the ED for more than 8 h. Access block cases include patients admitted to observation/short stay areas in accordance with established definitions [20].

Smaller values of these metrics are better for the patient and system—ambulances are back on the road sooner, and patients are closer to definitive diagnosis and treatment in hospital.

To assess the impact of COVID‐19, the study period was divided into three time periods:

- Pre–COVID‐19 (2018–2019);
- During COVID‐19 (2020–2021); and
- Post–COVID‐19 (2022).

COVID‐19 infections continue worldwide. In this analysis, COVID‐19 refers to the first wave of the pandemic, whose primary impact on the health system was in 2020 and to a lesser degree in 2021.

Ambulance ramping is a component of the public hospital performance framework in other jurisdictions although definitions vary; for example, ‘turnaround time’ in the Australian Capital Territory and ‘transfer of care’ in other states. POST and response time, as measured by QAS, only includes QAS Priority Category 1 and 2 patients; these categories are dispatch‐determined, often based on 000 calls. Category 3 and 4 patients have a different pathway and are often planned medical transfers. POST was trimmed to be more than 0 and less than 480 min, aligned with QAS definitions and the target is that ≥ 90% of patients transported by QAS to EDs to be offloaded within 30 min. POST was calculated in minutes as the difference between ambulance arrival at the ED and patient handover time stamps.

Given the impacts of COVID‐19 on hospital demand worldwide, the study time period was segmented to enable POST to be examined before, during and after the first wave of the pandemic. The relationship between the ambulance metrics response time and POST was also explored. In addition, we examined the relationship between POST and access block, including variations across COVID‐19 periods, and Australian Triage Scale (ATS) urgency levels. Finally, we explored the relationship between ambulance ramping incidence and ED length of stay, the duration between ED presentations and ED departure using the combined QAS and ED data. Identified relationships are discussed to support the selection of actionable strategies for improving patient flow.

We report our study as per the Strengthening the Reporting of Observational Studies in Epidemiology (STROBE) guidelines (Table S1).

### 2.2 Statistical Analysis

Continuous data were summarised as means with standard deviation (SD) or medians with interquartile ranges (IQRs), and categorical data as counts with proportions. The empirical cumulative distribution function was used to explain how the data were spread out. Ordinary least squares and error‐in‐variables regression models were used to investigate the relationship between response time and POST. An unbalanced multi‐way analysis of variance (ANOVA) was used to investigate how POST was related to triage category and access block by time period. A binomial generalised linear model was used to model the relationship between access block (yes = 1, no = 0) and POST in each group (pre–, during and post–COVID‐19) and each triage category. A Poisson generalised linear model with separate slopes of the hospitals through the origin was employed to examine the relationship between ambulance ramping incidents and ED length of stay. Where overdispersion was detected, a negative binomial generalised linear model was employed. All analyses were conducted using the R statistical software package (R version 4.4.0) [21].

### 2.3 Ethics Statement

An ethics exemption for this quality improvement study was granted by the Metro South Human Research Ethics Committee (EX/2022/QMS/89905), with the intent to publish the findings.

## 3 Results

### 3.1 Queensland Ambulance Service Data

Out of 3,227,933 ambulance records, we included 3,030,704 records after considering the inclusion criteria of Priority 1 (emergency) and Priority 2 (acute) ambulance 000 calls, and non‐negative POST capped at 480 min (8 h). POST ≤ 0 data were excluded as logically invalid with QAS definitions and most likely a data entry error, 72,363 (2.4%) records. Finally, we identified 2,571,476 ambulance records corresponding to the 25 hospitals in the ED data.

### 3.2 POST Comparison Between Pre–, During and Post–COVID‐19

POST descriptive statistics are presented in Table 1. The priority of cases was about evenly split between Priority 1 (1,397,250; 54.3%) and Priority 2 (1,174,226; 45.6%), and median POST were 24.1 min (IQR, 15.6–37.3 min) and 22.5 min (IQR, 14.7–34.3 min), respectively, for patients arriving at ED by ambulance.

**TABLE 1:** Patient off‐stretcher time by ambulance priority.

Priority	*N* (%)	Range, min	Median (IQR), min	Mean (SD), min
1	1,397,250 (54.3%)	0.017–480	24.1 (15.6–37.3)	33.4 (0.03)
2	1,174,226 (45.6%)	0.017–480	22.5 (14.7–34.3)	30.8 (0.03)

Abbreviations: IQR, interquartile range; SD, standard deviation.

POST by time period and by priority is compared in Table 2. The mean and median POST of Priority 1 patients were higher post–COVID‐19, at 40.5 min (SD, 0.07 min) and 27.6 min (IQR, 17.5–47.5 min), respectively. Similarly, for Priority 2 patients, the mean and median POST were 36.0 min (SD, 0.08 min) and 24.8 min (IQR, 16.1–41.1 min). Before the COVID‐19 pandemic, the mean POST for both priority categories was less than 30 min. However, during and after the COVID‐19 pandemic, the mean POST exceeded this target.

**TABLE 2:** Patient off‐stretcher time by time period and ambulance priority.

	Pre–COVID‐19	During COVID‐19	Post–COVID‐19
Priority 1			
*N*	503,003	549,962	344,285
Range, min	0.02–477.1	0.02–479.9	0.02–479.9
Median (IQR), min	19.8 (13.4–31.1)	23.2 (15.3–38.6)	27.6 (17.5–47.5)
Mean (SD), min	27.4 (0.04)	34.1 (0.05)	40.5 (0.07)
Priority 2			
*N*	470,267	503,464	200,495
Range, min	0.02–476.5	0.02–479.9	0.02–476.4
Median (IQR), min	19.5 (13.2–30.4)	21.8 (14.5–35.3)	24.8 (16.1–41.1)
Mean (SD), min	27.1 (0.04)	31.4 (0.05)	36.0 (0.08)

Abbreviations: COVID‐19, coronavirus disease 2019; IQR, interquartile range; SD, standard deviation.

Compared with pre‐pandemic performance, the difference in mean POST during and post–COVID‐19 was 6.7 min (95% confidence interval [CI], 6.5–6.9 min) and 13.1 min (95% CI, 12.9–13.3 min), respectively, for Priority 1, and 4.3 min (95% CI, 4.2–4.5 min) and 8.9 min (95% CI, 8.7–9.1 min), respectively, for Priority 2, and these differences between periods were all significant (*p* < 0.001).

### 3.3 POST Threshold Compliance

A comparison of POST compliance against QAS target of 30 min, along with 40‐ and 60‐min intervals, is presented in Table 3. In 2018, the highest POST threshold compliance was 355,585 (76%), followed by 363,327 (72%) and 373,718 (72%) both 2019 and 2020. The lowest POST threshold compliance within 30 min was 309,815 (57%) in 2022 (post–COVID‐19). Compliance across the time periods decreased from 718,912 (74%) pre–COVID‐19 to 694,633 (66%) during COVID‐19 and 309,815 (57%) post–COVID‐19, representing 8% and 17% declines, respectively. These figures are well below the 90% target, with shortfalls of 16%, 24% and 33%, for pre–COVID‐19, during COVID‐19 and post–COVID‐19, respectively.

**TABLE 3:** Patient off‐stretcher time (POST) compliance before, during and after COVID‐19.

Year	Total number of cases	POST compliance (%)		
		30 min	40 min	60 min
Pre–COVID‐19 (2018)	466,562	355,585 (76%)	400,135 (86%)	433,096 (93%)
Pre–COVID‐19 (2019)	506,708	363,327 (72%)	416,576 (82%)	459,550 (91%)
Overall	973,270	718,912 (74%)	816,711 (84%)	892,646 (92%)
During COVID‐19 (2020)	519,028	373,718 (72%)	431,289 (83%)	475,176 (92%)
During COVID‐19 (2021)	534,398	320,915 (60%)	387,442 (73%)	449,795 (84%)
Overall	1,053,426	694,633 (66%)	818,731 (78%)	924,971 (88%)
Post–COVID‐19 (2022)	544,780	309,815 (57%)	384,163 (71%)	454,916 (84%)
Overall	544,780	309,815 (57%)	384,163 (71%)	454,916 (84%)

Abbreviation: COVID‐19, coronavirus disease 2019.

**FIGURE 1:**
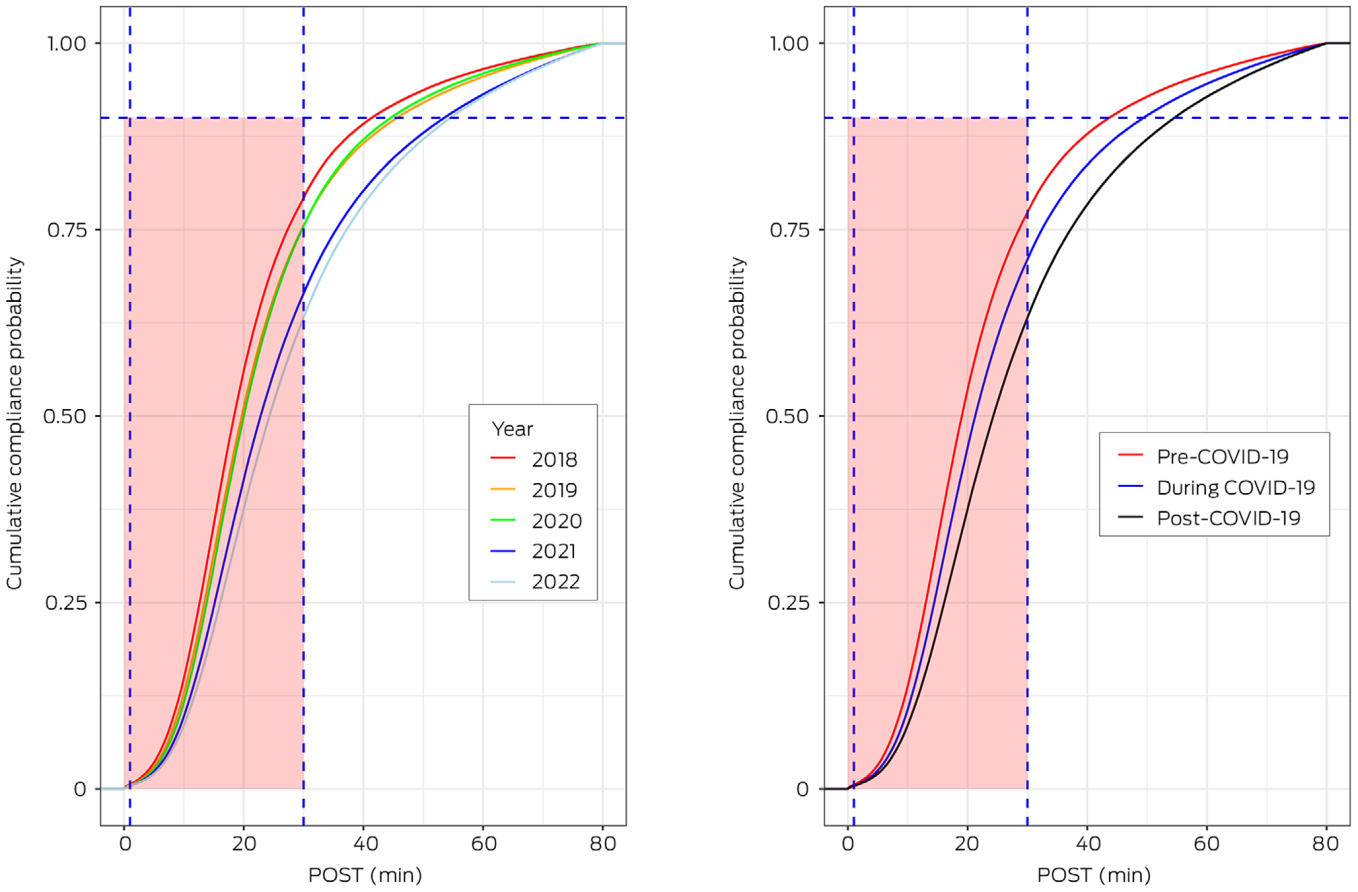
The empirical cumulative distribution functions of patient off‐stretcher time (POST) by year (left) and by COVID‐19 period (right). The red area of the box is a target of 90% being transferred from the ambulance to the emergency department within 30 min. COVID‐19, coronavirus disease 2019.

The empirical cumulative distribution function plots of POST pre–, during and post–COVID‐19 are shown in Figure 1. The vertical dashed line marks the 30‐min QAS target, and the horizontal line indicates the 90th percentiles of POST. The vertical differences between the two distributions for 30 min indicate the differences in compliance. Ambulance ramping increased by 6.1% pre–COVID‐19, 24.1% during COVID‐19 and 33.1% post–COVID‐19 [22, 23]. The horizontal difference between distributions represents the change in POST at a given compliance level. For instance, at 50% compliance in Figure 1 (right), the POST difference between pre– and post–COVID‐19 is shown as 24 min (post–COVID‐19) minus 19 min (pre–COVID‐19), indicating a 5‐min increase in POST during the post–COVID‐19 period compared with pre–COVID‐19. Note the axis for POST values was truncated at 80 min, where nearly 100% of patients have been offloaded.

### 3.4 Relationship Between POST and Response Time

**FIGURE 2:**
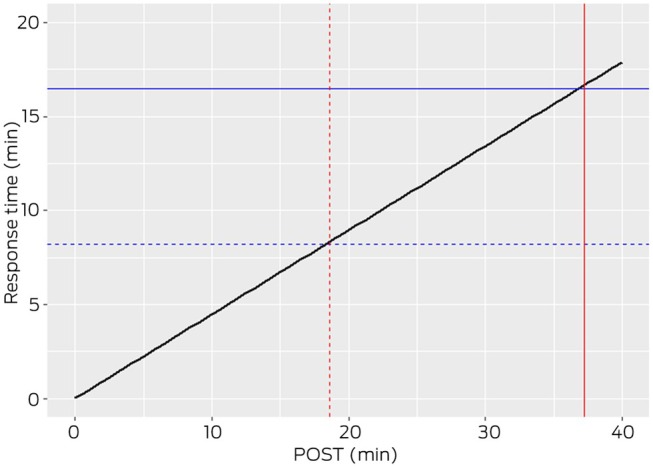
Relationship between response time and patient off‐stretcher time (POST). Blue lines: The percentile targets for response times (solid, 90th percentile; and dashed, 50th percentile). Red lines: Corresponding POSTs at the points where percentile targets intersect the regression line.

The relationship between POST and response time was investigated using both error‐in‐variables and ordinary least square regressions through the origin. The analysis revealed a positive relationship between metrics, described by the equation: Response Time = 0.45 × POST. In Figure 2, the blue dashed and solid lines represent QAS target percentiles for response time at the 50th (8.2 min) and 90th (16.5 min) percentiles, respectively [24]. Meanwhile, the red dashed and solid lines indicate POST at 18.2 and 36.7 min, respectively, corresponding to response time. Shorter response times are associated with shorter off‐stretcher times, releasing the ambulance back to the community sooner. Note the axes have been truncated to best illustrate the POST values corresponding to the current QAS response time targets.

### 3.5 QAS and Emergency Department Combined Data

We merged 3,203,737 ED records of road ambulance arrivals with QAS data using the event ID provided by the Statistical Service Branch of Queensland Health, yielding 2,818,401 combined data rows.

### 3.6 Relationship Between POST and Access Block

The percentage of access block between pre– and post–COVID‐19 periods increased from 10% to 17% but minimally changed between the pre– and during COVID‐19 periods (Table 4). Notably, irrespective of access block or non‐access block, POST consistently increased across the pre–, during and post–COVID‐19 periods. Pre–COVID‐19, the mean POST for patients without access block remained within the target (≤ 30 min). Post–COVID‐19, the mean POST values surpassed the target by 7 min for patients without access block, and by 19 min for patients with access block, respectively.

**TABLE 4:** Patient off‐stretcher time (POST) by pre‐defined time periods and access block group.

Periods (group)	*N* (%)	Median POST (IQR), min	Mean POST (SD), min
Pre–COVID‐19 access block	91,168 (10%)	23.5 (15.6–40.1)	34.5 (0.11)
Pre–COVID‐19 non‐access block	821,461 (90%)	19.4 (13.2–30.1)	26.6 (0.03)
During COVID‐19 access block	112,068 (11%)	28.1 (18.0–51.3)	42.9 (0.13)
During COVID‐19 non‐access block	876,145 (89%)	22.1 (14.8–35.8)	31.7 (0.03)
Post–COVID‐19 access block	87,757 (17%)	33.2 (20.6–59.6)	49.0 (0.16)
Post–COVID‐19 non‐access block	421,377 (83%)	25.6 (16.6–42.6)	36.9 (0.06)

Abbreviations: COVID‐19, coronavirus disease 2019, IQR, interquartile range; SD, standard deviation.

Pairwise comparisons of mean POST between different patient cohorts are illustrated in the Table S2. The mean POST for access block increased by 8.4 min from pre–COVID‐19 (34.5 min) to during COVID‐19 (42.9 min), by 6.1 min from during to post–COVID‐19 (49 min) and by 14.5 min from pre– to post–COVID‐19. These indicate a continuing increase in POST across the COVID‐19 periods (Table S2).

**FIGURE 3:**
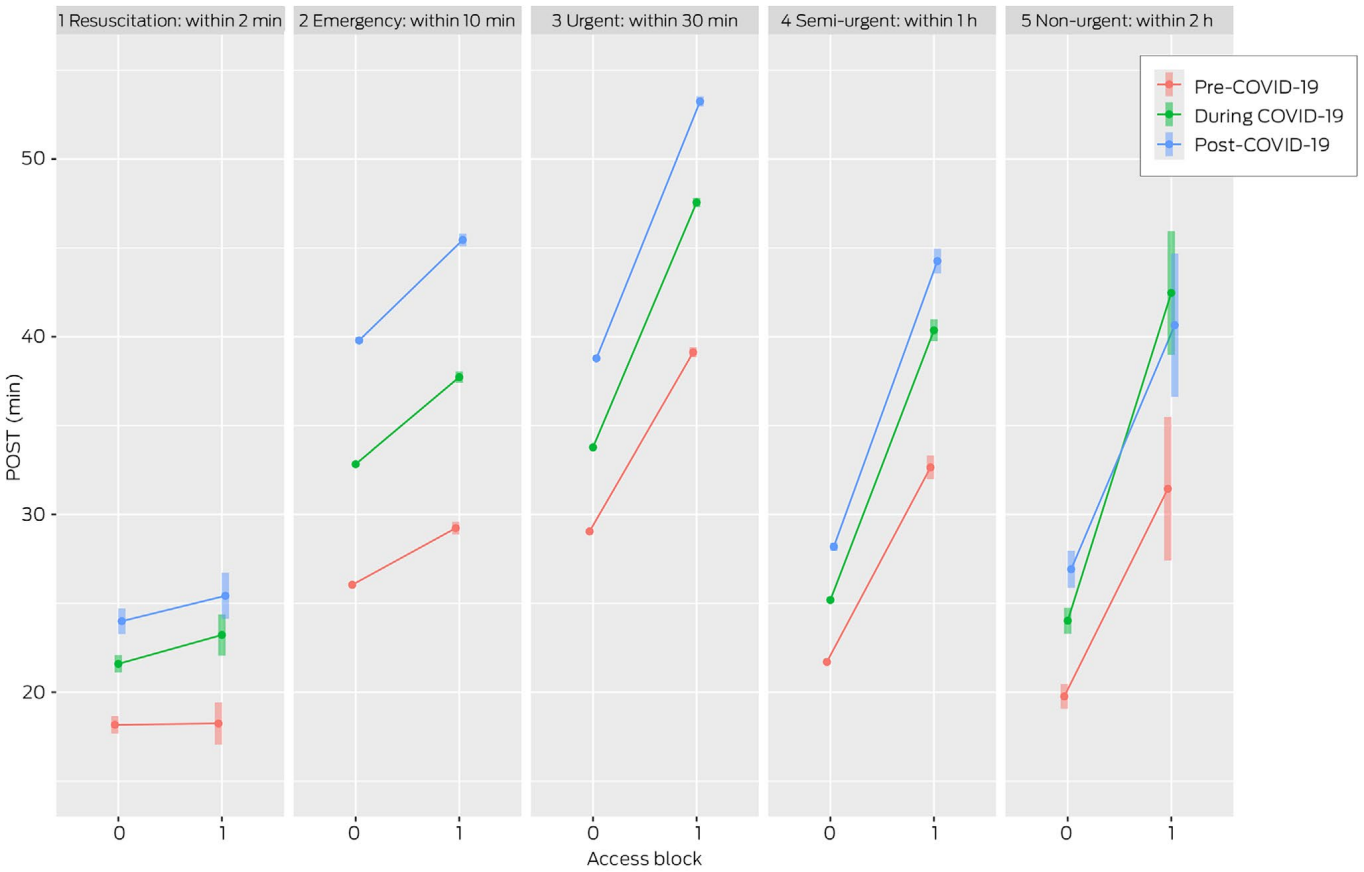
Patient off‐stretcher time (POST) by triage category and access block status by COVID‐19 periods. The 95% confidence intervals for the Australian Triage Scale categories 2–4 are very small due to the scale of the study. COVID‐19, coronavirus disease 2019.

The relationship between POST and triage category based on access block and COVID‐19 periods is presented in Figure 3. It can be observed that, with the exception of ATS1 patients, before the COVID‐19 pandemic, patients with access block had longer POST for all periods, and that, over time, patients experienced longer POST delays. For ATS1, regardless of access block status, POST remained within target compliance. However, during and post–COVID‐19, POST for those with access block were slightly higher compared with those without. In ATS2, POST was within target compliance pre–COVID‐19 for both access blocked and non‐access blocked patients, whereas during and post–COVID‐19, it exceeded the target for both groups. Conversely, for ATS4 and ATS5, POST for non‐access block was below 30 min, and for access block, it exceeded the target compliance. The worst POST performance was observed for ATS3 patients.

### 3.7 Statistical Modelling

**FIGURE 4:**
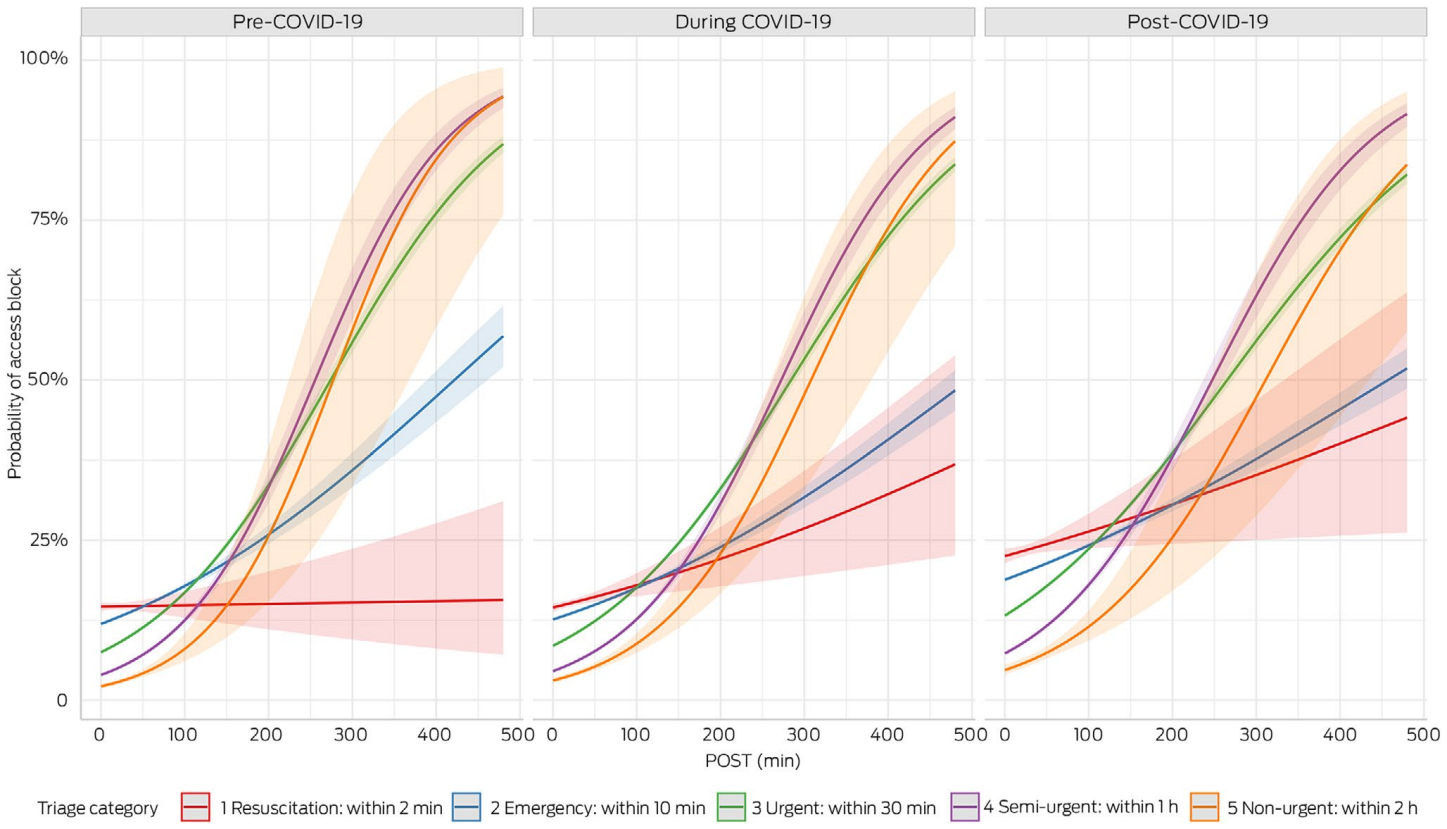
Relationship between patient off‐stretcher time (POST) and access block (shaded areas are 95% confidence intervals). The figure is based on a statistical model, and the predictions reflect the model's outputs. Therefore, the x‐axis extends to the maximum POST value of 480 min. COVID‐19, coronavirus disease 2019.

The relationship between access block and POST, categorised by triage levels during COVID‐19, is illustrated in Figure 4. Pre–COVID‐19, for ATS1, the odds ratio was nearly 1, indicating no significant change in access block incidence with varying POST levels. However, for ATS2–5, the odds ratios greater than 1 indicated that access block incidence rose with increasing POST. Furthermore, Figure 4 shows that during and post–COVID‐19, the probability of being access block increased consistently across ATS1, highlighting a notable rise in the likelihood of access block in this category over the specified periods.

The association between POST incidents exceeding the 30 min target and ED length of stay, assessed with a generalised linear model with negative binomial distribution to account for overdispersion, is illustrated in the Figure S1. For all study sites (coded by Australian Institute of Health and Welfare [AIHW] peer grouping), we observed an association between ED length of stay and POST breaches. A decrease in ED length of stay corresponds to a reduction in the number of cases in which the POST threshold was exceeded. The magnitude of the association is given by the incidence rate ratios. Hospitals were analysed using AIHW peer groupings, which classify hospitals with similar size, role and service profiles to enable fair and meaningful comparisons. For example, for every 1‐h decrease in ED length of stay, the number of POST breaches drops by 26% at principal referral–1 and 17% at principal referral–2. Across all hospitals, this reduction ranges from 2% (public acute group B–5) to 29% (principal referral–6).

## 4 Discussion

This work demonstrates that achieving the POST compliance target—transferring 90% of patients to the ED within 30 min of arrival—is challenging. Despite this, shorter off‐stretcher times, even beyond the 30‐min threshold, are associated with improved response times. The current POST target, a key performance indicator, was recommended over a decade ago by the 2012 Metropolitan Emergency Department Access Initiative (MEDAI) review [25]. Although targets should not be adjusted to make them achievable, they should be based on evidence or data‐driven and linked to patient outcomes or system impacts.

A positive association was identified between ambulance response time and POST: as POST increases, so does the ambulance response time (Figure 2). Ramping delays the return to service of ambulance crews and extends the time for ambulances to respond to new emergencies.

Statewide targets for ambulance response times are set at 8.2 and 16.5 min for the 50th and 90th percentiles, respectively [26]. There was a positive association between response time and POST; POST was about 18.2 min at the 50th percentile of response time and 36.7 min for the 90th percentile. It is also noted that the most urgent arrivals (ATS1 and ATS2 patients) have the same 30‐min ambulance offload target as less urgent arrivals. A shorter offload target for these patients may be prudent, especially given current policy concerning the maximum waiting time for ED medical assessment and treatment [27].

POST compliance is challenging due to the access block downstream from the ED preventing incoming patients. To reduce POST delays and improve ambulance services, efforts should focus on enhancing ward beds access and increasing hospital capacity.

POST has demonstrated a consistent upward trend, showing longer delays in patient handover and subsequent care initiation within the ED setting [9, 18]. Access block primarily results from patients categorised as ATS3–5, but is also evident in more urgent cases. We found that patients who experienced access block had longer POST, which confirms suggestions in the literature that access block increases ambulance delays [9]. This phenomenon is exacerbated because ambulance arrivals have higher admission rate than walk‐in patients [28], increasing access block's impact on ambulance operations.

There is a positive relationship between ED length of stay and ambulance ramping: improving ED length of stay is associated with reduced ambulance ramping, with varying effects across hospitals (Figure S1). The importance of minimising ED length of stay in mitigating ambulance ramping occurrences is emphasised from these results and supported by the literature [10, 29]. Hospitals and emergency services may consider strategies to streamline ED processes, aiming to expedite patient care and reduce waiting times, which could alleviate ambulance ramping [30].

The next crucial step is to link ambulance and ED data with inpatient data. This will enable evaluation of patient outcomes, such as inpatient length of stay and mortality, and determine if prolonged off‐stretcher times are associated with poorer clinical outcomes and informing more effective inpatient interventions.

### 4.1 Limitations

The study's primary limitation is that the ongoing, evolving nature of the COVID‐19 pandemic may act as a confounding factor. The fluctuating impact of COVID‐19 on hospital admissions, staffing and policies could skew findings on POST, ambulance ramping and access block, making it difficult to isolate the effects of the pandemic from other operational variables in patient flow. Another limitation is the study's focus on ambulance arrivals, acknowledging that self‐presenting ED patients also experience access block. We used hourly aggregated data to compare flow metrics, but acknowledge that a more detailed time‐lag analysis could further clarify causality. Relationships between key metrics were investigated directly where possible and inferred indirectly in some cases. Our study, while data‐driven and focused on individual‐level associations, does not fully capture the broader, system‐level and cumulative nature of hospital crowding. The observed relationships between POST, access block and response time may reflect underlying operational pressures beyond our study's scope.

## 5 Conclusion

Meeting the POST compliance target of transferring 90% of ambulance patients to ED care within 30 min is becoming increasingly challenging, with performance dropping in recent years. The study found an association between POST and ambulance response time, and between POST and access block, hence linking these ambulance metrics with the adverse patient outcomes associated with access block. Access block affects patients across all triage categories, causing congestion, particularly for ambulance arrivals, who have higher admission rates.

## Author Contributions

**Hwan‐Jin Yoon:** conceptualisation; data curation; formal analysis; methodology; visualisation; writing (original draft); writing (review and editing). **Justin Boyle:** conceptualisation; data curation; investigation; supervision; writing (original draft); writing (review and editing). **Ibrahima Diouf:** writing (review and editing). **Emma Bosley:** writing (review and editing). **Andrew Staib:** writing (review and editing). **Vahid Riahi:** writing (review and editing). **Hamed Hassanzadeh:** writing (review and editing). **Mahnaz Samadbeik:** writing (review and editing). **Clair Sullivan:** writing (review and editing). **Sankalp Khanna:** writing (review and editing). **James F. Lind:** writing (review and editing).

## Funding

The authors acknowledge the support of this work from the Emergency Medicine Foundation, the Queensland Ambulance Service and Queensland Health.

## Conflicts of Interest

The authors declare no conflicts of interest.

## Supporting information


**Data S1:** mja270141‐sup‐0001‐supinfo.pdf.

## Data Availability

Data relating to the analysis of ambulance and hospital records are unable to be shared due to ethics and regulatory limitations.
