# Supplementary material for: The Association Between Access Block And Ambulance Ramping, And The Impact of COVID‐19: A Retrospective Observational Cohort Study of 25 Queensland Hospitals
Source: Med J Aust. 2026 Feb 10;224(2):e70141. doi: 10.5694/mja2.70141 (PMC12888069; doi:10.5694/mja2.70141)
Supplement: Supplementary file 1 — Data S1: mja270141‐sup‐0001‐supinfo.pdf. [file MJA2-224-0-s001.pdf]

MJA
The Medical Journal of Australia

Supporting Information

Supplementary material

This appendix was part of the submitted manuscript and has been peer reviewed.
It is posted as supplied by the authors.

Appendix to: H. J. Yoon, J. Boyle, I. Diouf, et al., "The Association Between Access Block And Ambulance Ramping, And The Impact of COVID-19: A Retrospective Observational Cohort Study of 25 Queensland Hospitals", *Medical Journal of Australia* 224 (2026); doi: 10.5694/mja2.70141.

TABLE S1. Strengthening the Reporting of Observational Studies in Epidemiology (STROBE) Statement—checklist of items that should be included in reports of *cohort studies*.

	Item No	Recommendation
**Title and abstract**	1	(*a*) Indicate the study's design with a commonly used term in the title or the abstract - check
		(*b*) Provide in the abstract an informative and balanced summary of what was done and what was found - check
**Introduction**		
Background/rationale	2	Explain the scientific background and rationale for the investigation being reported - check
Objectives	3	State specific objectives, including any prespecified hypotheses - check
**Methods**		
Study design	4	Present key elements of study design early in the paper - check
Setting	5	Describe the setting, locations, and relevant dates, including periods of recruitment, exposure, follow-up, and data collection – check
Participants	6	(*a*) Give the eligibility criteria, and the sources and methods of selection of participants. Describe methods of follow-up
- N/A		(*b*) For matched studies, give matching criteria and number of exposed and unexposed
Variables	7	Clearly define all outcomes, exposures, predictors, potential confounders, and effect modifiers. Give diagnostic criteria, if applicable - check
Data sources/ measurement	8*	For each variable of interest, give sources of data and details of methods of assessment (measurement). Describe comparability of assessment methods if there is more than one group - check
Bias	9	Describe any efforts to address potential sources of bias – N/A
Study size	10	Explain how the study size was arrived at - check
Quantitative variables	11	Explain how quantitative variables were handled in the analyses. If applicable, describe which groupings were chosen and why - check
Statistical methods	12	(*a*) Describe all statistical methods, including those used to control for confounding
		(*b*) Describe any methods used to examine subgroups and interactions
- check		(*c*) Explain how missing data were addressed
		(*d*) If applicable, explain how loss to follow-up was addressed
		(*e*) Describe any sensitivity analyses
**Results**		
Participants	13*	(a) Report numbers of individuals at each stage of study—eg numbers potentially eligible, examined for eligibility, confirmed eligible, included in the study, completing follow-up, and analysed
- N/A		(b) Give reasons for non-participation at each stage
		(c) Consider use of a flow diagram
Descriptive data	14*	(a) Give characteristics of study participants (eg demographic, clinical, social) and information on exposures and potential confounders

		(b) Indicate number of participants with missing data for each variable of interest
- N/A		(c) Summarise follow-up time (eg, average and total amount)
Outcome data	15*	Report numbers of outcome events or summary measures over time - check
Main results	16	(*a*) Give unadjusted estimates and, if applicable, confounder-adjusted estimates and their precision (eg, 95% confidence interval). Make clear which confounders were adjusted for and why they were included
- check		(*b*) Report category boundaries when continuous variables were categorized
		(*c*) If relevant, consider translating estimates of relative risk into absolute risk for a meaningful time period
Other analyses	17	Report other analyses done—eg analyses of subgroups and interactions, and sensitivity analyses - check
**Discussion**		
Key results	18	Summarise key results with reference to study objectives - check
Limitations	19	Discuss limitations of the study, taking into account sources of potential bias or imprecision. Discuss both direction and magnitude of any potential bias - check
Interpretation	20	Give a cautious overall interpretation of results considering objectives, limitations, multiplicity of analyses, results from similar studies, and other relevant evidence<br>- check
Generalisability	21	Discuss the generalisability (external validity) of the study results - check
**Other information**		
Funding	22	Give the source of funding and the role of the funders for the present study and, if applicable, for the original study on which the present article is based - check

*Give information separately for exposed and unexposed groups.

Note: An Explanation and Elaboration article discusses each checklist item and gives methodological background and published examples of transparent reporting. The STROBE checklist is best used in conjunction with this article (freely available on the Web sites of *PLoS Medicine* at http://www.plosmedicine.org/, *Annals of Internal Medicine* at http://www.annals.org/, and *Epidemiology* at http://www.epidem.com/). Information on the STROBE Initiative is available at http://www.strobe-statement.org.

TABLE S2. Pairwise comparisons of mean patient off-stretcher time (SD) between different patient cohorts.

Periods (Group)	Pre		During		Post	
	Non-access block	Access block	Non-access block	Access block	Non-access block	Access block
Pre-COVID-19 non-access block	26.6	***	***	***	***	***
Pre-COVID-19 access block	–7.9 (0.11)	34.5	***	***	***	***
During COVID-19 non-access block	–5.1 (0.05)	2.8 (0.11)	31.7	***	***	***
During COVID-19 access block	–16.3 (0.10)	–8.4 (0.14)	–11.2 (0.10)	42.9	***	***
Post-COVID-19 non-access block	–10.3 (0.06)	–2.4 (0.12)	–5.2 (0.06)	6.0 (0.10)	36.9	***
Post-COVID-19 access block	–22.4 (0.11)	–14.5 (0.15)	–17.3 (0.11)	–6.1 (0.14)	–12.1 (0.12)	49.0

Note: Estimates along the diagonal, *P* value in the upper triangle (* < 0.05, ** < 0.01, *** < 0.001), and the difference in mean (SD) in the lower triangle. SD, standard deviation; COVID-19, coronavirus disease 2019.

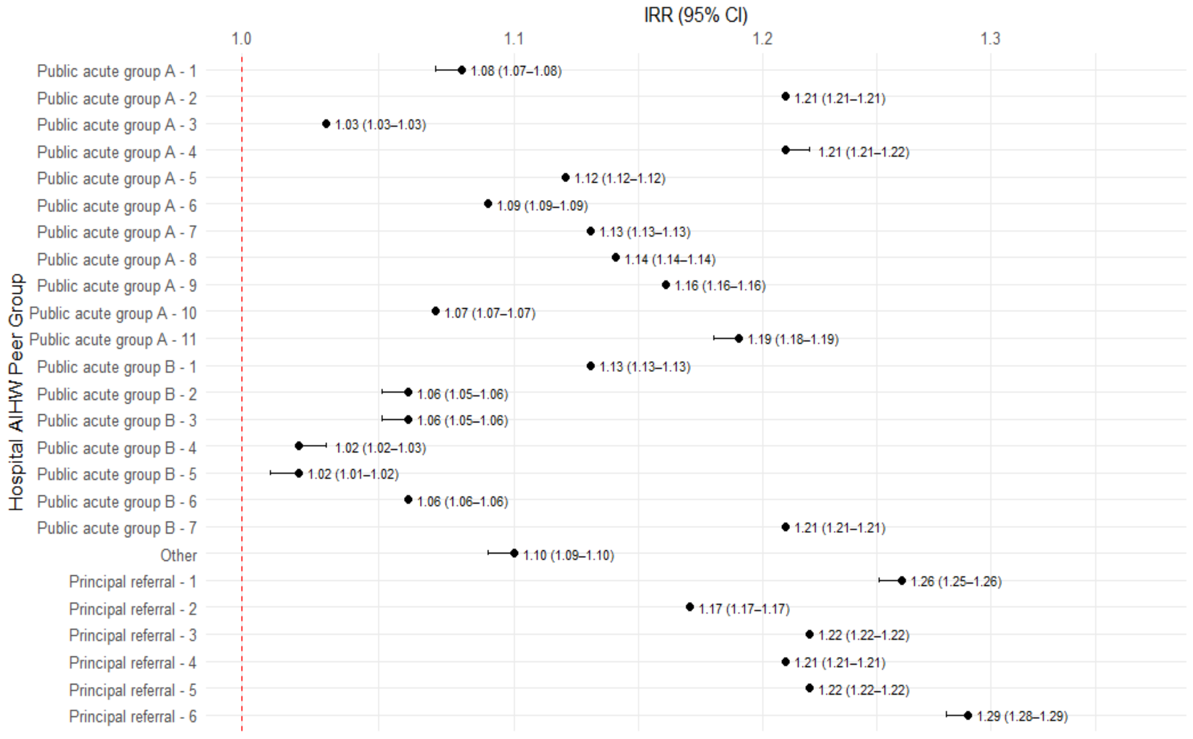

FIGURE S1. Association between emergency department length of stay and the number of cases where the patient off-stretcher time threshold was exceeded. IRR, incidence rate ratio; CI, confidence interval; AIHW, Australian Institute of Health and Welfare.
